# The Effect of *tert*-Butyl Hydroperoxide-Induced Oxidative Stress on Lean and Steatotic Rat Hepatocytes *In Vitro*

**DOI:** 10.1155/2014/752506

**Published:** 2014-03-31

**Authors:** Otto Kučera, René Endlicher, Tomáš Roušar, Halka Lotková, Tomáš Garnol, Zdeněk Drahota, Zuzana Červinková

**Affiliations:** ^1^Department of Physiology, Charles University in Prague-Faculty of Medicine in Hradec Králové, Šimkova 870, 500 38 Hradec Králové, Czech Republic; ^2^Department of Anatomy, Charles University in Prague-Faculty of Medicine in Hradec Králové, Šimkova 870, 500 38 Hradec Králové, Czech Republic

## Abstract

Oxidative stress and mitochondrial dysfunction play an important role in the pathogenesis of nonalcoholic fatty liver disease and toxic liver injury. The present study was designed to evaluate the effect of exogenous inducer of oxidative stress (*tert*-butyl hydroperoxide, tBHP) on nonfatty and steatotic hepatocytes isolated from the liver of rats fed by standard and high-fat diet, respectively. In control steatotic hepatocytes, we found higher generation of ROS, increased lipoperoxidation, an altered redox state of glutathione, and decreased ADP-stimulated respiration using NADH-linked substrates, as compared to intact lean hepatocytes. Fatty hepatocytes exposed to tBHP exert more severe damage, lower reduced glutathione to total glutathione ratio, and higher formation of ROS and production of malondialdehyde and are more susceptible to tBHP-induced decrease in mitochondrial membrane potential. Respiratory control ratio of complex I was significantly reduced by tBHP in both lean and steatotic hepatocytes, but reduction in NADH-dependent state 3 respiration was more severe in fatty cells. In summary, our results collectively indicate that steatotic rat hepatocytes occur under conditions of enhanced oxidative stress and are more sensitive to the exogenous source of oxidative injury. This confirms the hypothesis of steatosis being the first hit sensitizing hepatocytes to further damage.

## 1. Introduction

Nonalcoholic fatty liver disease (NAFLD) is the most common chronic liver disease in the USA and the Western world, and the prevalence of NAFLD has recently dramatically increased and it seems that this trend will continue [1, 2]. There is accumulating evidence that hepatic mitochondrial dysfunction and oxidative stress play an important role in the pathogenesis of NAFLD [3]. There are several mitochondrial abnormalities associated with NAFLD including ultrastructural lesions, depletion of mitochondrial DNA, decreased activity of respiratory chain complexes, and impaired mitochondrial *β*-oxidation [4–6]. Oxidative stress and resulting altered redox balance seem to be crucial in the pathogenesis of steatosis, steatohepatitis, and fibrosis [7]. Mitochondrial dysfunction and increased oxidative stress are closely related. Mitochondrial dysfunction can directly lead to the excess of reactive oxygen species (ROS) production [8]. On the other hand, oxidative stress may be a cause of mitochondrial dysfunction [9]. Altered hepatic redox status was proofed not only in the advanced forms of NAFLD, such as nonalcoholic steatohepatitis (NASH), but also in simple steatosis [10]. Enhanced oxidative stress is thought to belong to second hits that participate in the progression of simple steatosis to steatohepatitis [11]. Increased generation of free radicals and other highly reactive substances results from fat accumulation in the liver, particularly due to direct toxicity of fatty acids, increased peroxisomal and microsomal oxidation of fatty acids, and mitochondrial dysfunction [12, 13]. Other sources of oxidative stress, such as xenobiotic-induced hepatotoxicity, may very likely potentiate progression of simple steatosis to steatohepatitis. In previous experiments, we have found in accordance to others that rat steatotic hepatocytes exert higher sensitivity to the acute injury caused by hepatotoxins* in vivo* [14–16] and* in vitro* [17]. NAFLD, including simple steatosis, predisposes the liver to the increased risk of hepatotoxicity [18].

Oxidative stress is one of the general mechanisms involved in hepatotoxicity. Free radicals initiate lipid peroxidation of polyunsaturated fatty acids in membranes which results in membrane disruption, formation of reactive aldehydes, and depletion of cellular storage of reduced glutathione (GSH).* tert*-Butyl hydroperoxide (t-BHP) is commonly used as a model substance for evaluation of mechanisms of cellular alterations resulting from oxidative stress in cells and tissues. There are two pathways by which tBHP is metabolized; both of them induce oxidative stress. The first, provided by cytochrome P450, leads to production of peroxyl and alkoxyl radicals [19]. These radicals initiate lipoperoxidation of membrane phospholipids with subsequent alterations to membrane fluidity and permeability. The other pathway employs glutathione peroxidase. tBHP is detoxified to* tert*-butanol and GSH is depleted by oxidation to its disulphide form (GSSG) [20]. Lipoperoxidation, depletion of GSH, and the onset of mitochondrial permeability transition (MPT) are general mechanisms involved in cell injury caused by oxidative stress. Thus usage of exogenous inducer of oxidative stress, such as tBHP, may simulate situation of augmented oxidative stress in fatty hepatocytes and helps us to understand particular mechanisms in the pathogenesis of NAFLD.

The present study was designed to determine and to compare tBHP-induced oxidative stress in isolated lean and steatotic rat hepatocytes in primary culture. For our model of cell injury, we used tBHP, a short-chain organic hydroperoxide, which is an analogue of the products of lipoperoxidation formed during oxidative stress and may mimic oxidative stress in human diseases.

## 2. Material and Methods

### 2.1. Chemicals

Medium William's E (without phenol red), foetal bovine serum, penicillin, streptomycin, and glutamine were purchased from PAN BIOTECH GmbH (Aidenbach, Germany). Collagenase (Collagenase NB 4 Standard Grade from* Clostridium histolyticum*) was obtained from SERVA Electrophoresis GmbH (Heidelberg, Germany), insulin and glucagon (Actrapid, Novo Nordisk A/S, Bagsvaerd, Denmark) and prednisolon (Merck KGaA, Darmstadt, Germany) were from the suppliers mentioned in brackets. Type I collagen, trypan blue,* tert*-butyl hydroperoxide solution (Cat. number: 458139), and all other chemicals were purchased from Sigma-Aldrich (St. Louis, MO).

### 2.2. Animals

Male albino Wistar rats (BioTest, Konárovice, Czech Republic) were housed at 23 ± 1°C, 55 ± 10% relative humidity, air exchange of 12–14 times/h, and 12 h light-dark cycle periods (6:00 h to 18:00 h). Rats used for isolation of nonsteatotic hepatocytes (NH) were fed* ad libitum* a standard pelleted diet (ST-1 diet, Velaz, Prague, Czech Republic; 10% energy fat, 30% energy proteins, and 60% energy saccharides) for 6 weeks. For isolation of fatty hepatocytes (SH), animals were fed high-fat gelled diet (71% energy fat, 18% energy proteins, and 11% energy saccharides) for 6 weeks [21]. The animals had free access to tap water. All animals received care according to the guidelines set by the Animal-Welfare Body of the Charles University, Prague, Czech Republic, and the International Guiding Principles for Biomedical Research Involving Animals. All animal experiments were approved by the committee mentioned above and by the Ministry of Education, Youth, and Sports (authorisation reference number 1315-28572/2012-30).

### 2.3. Hepatocyte Isolation, Cultivation, and Treatment

Under ether anaesthesia, hepatocytes were isolated by two-step collagenase perfusion from rat liver [22] with viability higher than 90% (trypan blue exclusion test). Isolated hepatocytes were suspended in supplemented William's E medium with 6% foetal bovine serum [17] and plated in collagen-coated Petri dishes (60 mm, 2 × 10^6^ cells/dish) or 12- (4 × 10^5^ cells/well) and 96-well (3 × 10^4^ cells/well) plates. Hepatocytes were allowed to establish monolayer in a humidified atmosphere containing 95% air and 5% CO_2_ at 37°C for 2 h. Then the medium was replaced with a fresh supplemented medium without foetal bovine serum according to the experimental protocol. Nonfatty and steatotic hepatocytes in primary cultures were incubated with tBHP (0.01–1 mmol/L) for up to 60 min. The other portion of hepatocytes was preincubated in William's E medium with or without 5 *μ*M trifluoperazine (TFP) for 30 min and then exposed to tBHP (0.25; 0.375; 0.5 mmol/L) with or without 5 *μ*M TFP for further 30 min (Table 1). After incubation, the medium was collected and cells were harvested for the required assays. Suspension of digitonin-permeabilized hepatocytes pretreated with 0.25 mM tBHP for 5 min was used for evaluation of mitochondrial respiration.

### 2.4. Cytotoxicity Assays

Plasma membrane integrity of cultured hepatocytes was determined by lactate dehydrogenase (LDH) activity in the medium using a commercial kit from DiaSys (Holzheim, Germany). Cell viability was also evaluated by measurement of the activity of cellular dehydrogenases using Cell Proliferation Reagent WST-1 (Roche Diagnostics, Mannheim, Germany) [23].

### 2.5. Estimation of GSH to Total Glutathione Ratio and Production of Reactive Oxygen Species (ROS) and Malondialdehyde (MDA)

For assessment of intracellular GSH to total glutathione ratio [GSH/(2 × GSSG + GSH)], cells were firstly lysed and harvested and then GSH and GSSG were measured by spectrofluorometric assay based on the reaction between GSH and* o*-phthalaldehyde (*λ* (exc) = 340 nm, pH 6.0) [24].

The production of ROS was evaluated using 5- and 6-chloromethyl-2′,7′-dichlorodihydrofluorescein diacetate (CM-H2DCFDA; Molecular Probes, Eugene, OR). After incubation, the cells were washed in William's E medium and loaded by 1 *μ*M CM-H2DCFDA for 45 min and then rinsed again in nonsupplemented William's E medium. Then fluorescence intensity was measured for 40 min (TECAN Infinite M200, Tecan Austria GmbH, Grödig, Austria, excitation and emission wavelength of 485 and 535 nm, resp.). Results are expressed in percent where control nonsteatotic hepatocytes are 100% of fluorescence intensity difference (at 40′ minus at 0′).

Secondary end product of lipoperoxidation MDA in culture medium was determined by the assessment of thiobarbituric acid reactive substances [25].

### 2.6. Production of Albumin

Functional capacity of cultured hepatocytes was evaluated by the amount of albumin secreted into the culture medium using Rat Albumin ELISA Quantification Kit (Bethyl Laboratories, Montgomery, TX).

### 2.7. Visualization of Mitochondrial Membrane Potential (MMP)

MMP was depicted using hepatocyte uptake of JC-1 (Molecular Probes, Inc., Oregon, USA), a cationic carbocyanine dye that accumulates in mitochondria according to its membrane potential. At low membrane potential, JC-1 exerts a green fluorescence (*λ*em 525 nm). At higher potentials, JC-1 forms red-fluorescent “J-aggregates” (*λ*em 590 nm). Hepatocytes were incubated with 10 *μ*M JC-1 (dissolved in William's E medium) in humidified atmosphere containing 95% air and 5% CO_2_ at 37°C for 30 min, then the cells were washed twice with fresh media. MMP was visualized using fluorescence microscope Olympus IX51 (Olympus, Japan) equipped with the digital camera Olympus E600 (Olympus, Japan). Results are expressed as percentage of cells containing mitochondria with high membrane potential.

### 2.8. Measurement of Oxygen Uptake by Isolated Hepatocytes

Oxygen consumption was measured using a High Resolution Oxygraph 2K (OROBOROS INSTRUMENTS GmbH, Innsbruck, Austria). Digitonin-permeabilized (10 *μ*g/mL) hepatocytes (125,000/mL) were incubated in 2 mL of K^+^-medium [26] at 30°C. State 4 respiration (10 mM glutamate + 2.5 mM malate) and state 3 respiration (10 mM glutamate + 2.5 mM malate + 1.5 mM ADP) were measured and respiratory control ratio (RCR, ratio of O_2_ consumption rate in state 3 to state 4) was calculated. Oxygen uptake at state 3 and state 4 is expressed as pmoles oxygen per second per million cells. For evaluation of oxygen uptake OROBOROS software (DatLab 3.1; OROBOROS INSTRUMENTS GmbH) was used.

### 2.9. Statistical Analysis

Experiments were performed at least three times using different isolations of hepatocytes. Due to high interexperiment variability, the data analyses were conducted by experiment. The results are expressed as means ± SD of a single representative experiment. After testing the normality, statistical analysis was performed by one-way analysis of variance (GraphPad Prism 6.01, GraphPad Software, Inc., La Jolla, CA). When significance was detected, Tukey-Kramer's post hoc test was used for comparisons between the different groups. *P* < 0.05 was considered to be statistically significant.

## 3. Results

### 3.1. Characteristics of Control Lean and Fatty Hepatocytes

**Figure 1:**
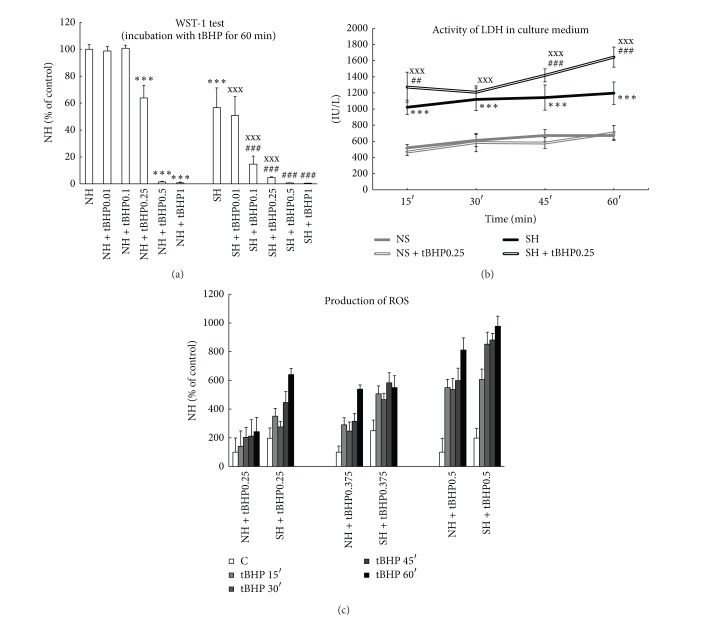
(a) WST-1 test of nonsteatotic (NH) and steatotic rat hepatocytes (SH) in primary culture treated with 0.01–1 mM tBHP (NH + tBHP; SH + tBHP) for 60 min. The values are means ± SD (*n* = 8). Results are expressed in percent where 100% is the activity of cellular dehydrogenases in control NH. ***P < 0.001 versus control NH; ^###^ *P* < 0.001 versus control SH; ^xxx^ *P* < 0.001 versus corresponding NH + tBHP. (b) Time course of LDH activity (IU/L) in media of lean (NH) and steatotic rat hepatocytes (SH) in primary cultures treated with 0.25 mM tBHP (NH + tBHP; SH + tBHP) for up to 60 min. The values are means ± SD (*n* = 6). ***P < 0.001 versus control NH; ^##^ *P* < 0.01 and ^###^ *P* < 0.001 versus control SH at corresponding time; ^xxx^ *P* < 0.001 versus NH + tBHP at corresponding time. (c) Time and concentration course of ROS generation (CM-H2DCFDA) in nonfatty (NH) and steatotic rat hepatocytes (SH) in primary culture treated with 0.25–0.5 mM tBHP (NH + tBHP; SH + tBHP) for up to 60 min. The values are means ± SD (*n* = 8). Results are expressed in percent where 100% is production of ROS by control NH for each concentration of tBHP. *P* values are not shown.

**Figure 2:**
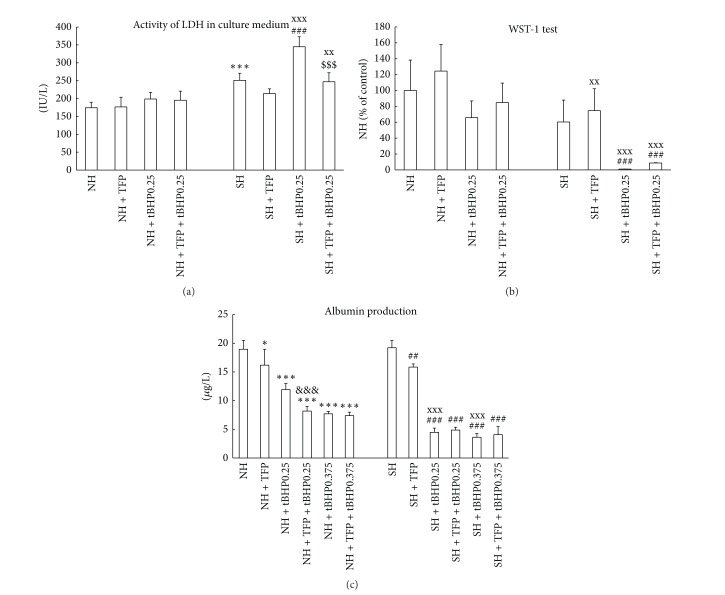
(a) LDH activity (IU/L) in medium of lean (NH) and steatotic rat hepatocytes (SH) in primary culture treated with 5 *μ*M TFP (NH + TFP; SH + TFP), with 0.25 mM tBHP (NH + tBHP; SH + tBHP) or with 5 *μ*M TFP and 0.25 mM tBHP (NH + TFP + tBHP; SH + TFP + tBHP). Cells were exposed to tBHP for a period of 30 min. The values are means ± SD (*n* = 6). ***P < 0.001 versus control NH; ^###^ *P* < 0.001 versus control SH; ^xx^ *P* < 0.01 and ^xxx^ *P* < 0.001 versus corresponding group in lean hepatocytes; ^$$$^ *P* < 0.001 versus SH + tBHP. (b) WST-1 test of nonsteatotic (NH) and steatotic rat hepatocytes (SH) in primary culture treated with 5 *μ*M TFP (NH + TFP; SH + TFP), with 0.25 mM tBHP (NH + tBHP; SH + tBHP), or with 5 *μ*M TFP and 0.25 mM tBHP (NH + TFP + tBHP; SH + TFP + tBHP). Cells were exposed to tBHP for 30 min. The values are means ± SD (*n* = 6). Results are expressed in percent where 100% is the activity of cellular dehydrogenases in control NH. ^###^ *P* < 0.001 versus control SH; ^xx^ *P* < 0.01 and ^xxx^ *P* < 0.001 versus corresponding group in lean hepatocytes. (c) Concentration of albumin (*μ*g/L) in culture medium of nonsteatotic (NH) and steatotic rat hepatocytes (SH) treated with 5 *μ*M TFP (NH + TFP; SH + TFP), with 0.25/0.375 mM tBHP (NH + tBHP; SH + tBHP), or with 5 *μ*M TFP and 0.25/0.375 mM tBHP (NH + TFP + tBHP; SH + TFP + tBHP). Cells were exposed to tBHP for 30 min. The values are means ± SD (*n* = 5). *P < 0.05 and ***P < 0.001 versus control NH; ^&&&^ *P* < 0.001 versus NH + tBHP0.25; ^##^ *P* < 0.01 and ^###^ *P* < 0.001 versus control SH; ^xxx^ *P* < 0.001 versus corresponding group in lean hepatocytes.

**Figure 3:**
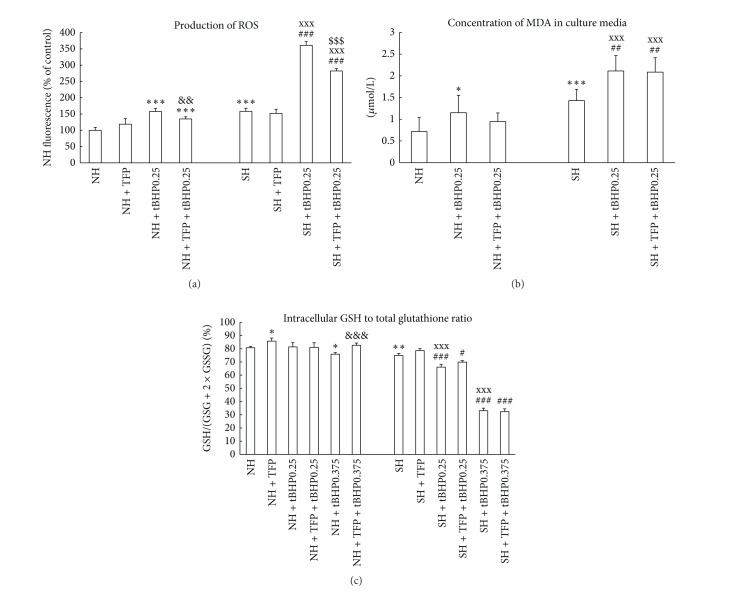
(a) Production of ROS (CM-H2DCFDA) in nonsteatotic (NH) and steatotic rat hepatocytes (SH) treated with 5 *μ*M TFP (NH + TFP; SH + TFP), with 0.25 mM tBHP (NH + tBHP; SH + tBHP), or with 5 *μ*M TFP and 0.25 mM tBHP (NH + TFP + tBHP; SH + TFP + tBHP). Cells were exposed to tBHP for 30 min. The values are means ± SD (*n* = 8). Results are expressed in percent where 100% is production of ROS by control NH. ***P < 0.001 versus control NH; ^&&^ *P* < 0.01 versus NH + tBHP; ^###^ *P* < 0.001 versus control SH; ^xxx^ *P* < 0.001 versus corresponding group in lean hepatocytes; ^$$$^ *P* < 0.001 versus SH + tBHP. (b) Concentration of MDA (*μ*mol/L) in culture media of nonsteatotic (NH) and steatotic rat hepatocytes (SH) treated with 0.25 mM tBHP (NH + tBHP; SH + tBHP) or with 5 *μ*M TFP and 0.25 mM tBHP (NH + TFP + tBHP; SH + TFP + tBHP). Cells were exposed to tBHP for 30 min. The values are means ± SD (*n* = 6). *P < 0.05 and ***P < 0.001 versus control NH; ^##^ *P* < 0.01 versus control SH; ^xxx^ *P* < 0.001 versus corresponding group in lean hepatocytes. (c) Intracellular GSH to total glutathione ratio in nonsteatotic (NH) and steatotic rat hepatocytes (SH) treated with 5 *μ*M TFP (NH + TFP; SH + TFP), with 0.25 mM tBHP (NH + tBHP; SH + tBHP), or with 5 *μ*M TFP and 0.25 mM tBHP (NH + TFP + tBHP; SH + TFP + tBHP). Cells were exposed to tBHP for 30 min. The values are means ± SD (*n* = 5). Results are expressed in percent of GSH from total glutathione. *P < 0.05 and **P < 0.01 versus control NH; ^&&&^ *P* < 0.001 versus NH + tBHP0.375; ^#^ *P* < 0.05 and ^###^ *P* < 0.001 versus control SH; ^xxx^ *P* < 0.001 versus corresponding group in lean hepatocytes.

**Figure 4:**
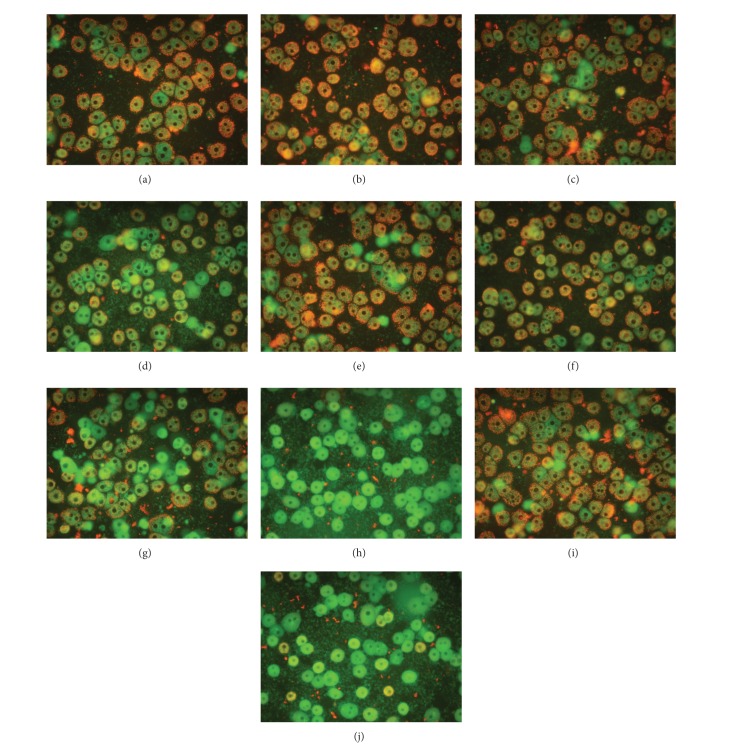
Visualization of changes in mitochondrial membrane potential using mitochondria specific fluorescent probe JC-1. Mitochondria with intact membrane potential concentrates JC-1 into aggregates (J-aggregates, red fluorescence at 590 nm), whereas deenergized mitochondria cannot concentrate JC-1 (green fluorescence at 530 nm). Microphotographs of nonsteatotic and fatty rat hepatocytes cultured in William's E medium (control, (a) and (b), resp.) with tBHP at concentration of 0.25 mmol/L ((c) and (d), resp.) and 0.375 mmol/L ((g) and (h), resp.) or with 5 *μ*M TFP and 0.25 mM ((e) and (f), resp.) or 5 *μ*M TFP and 0.375 mM tBHP ((i) and (j), resp.). Cells were exposed to tBHP for a period of 30 min. Magnification 400x.

**Figure 5:**
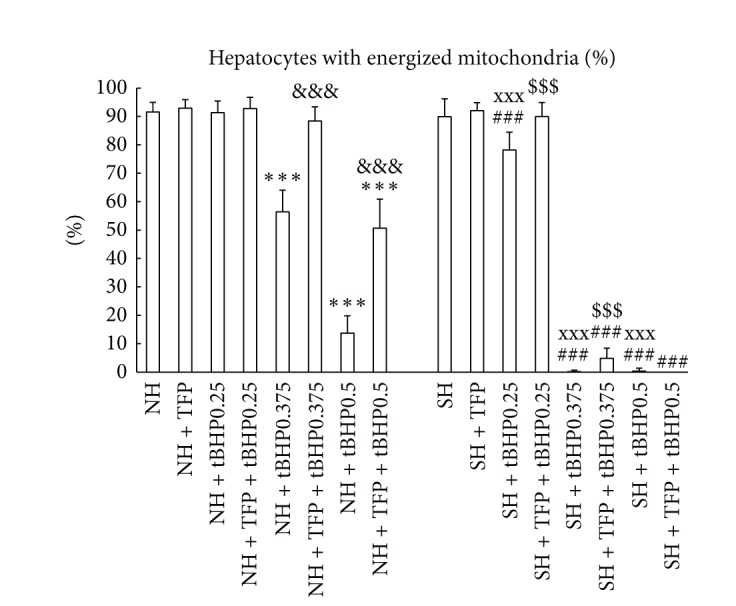
Percentage of nonsteatotic (NH) and steatotic rat hepatocytes (SH) with energized mitochondria treated with 5 *μ*M TFP (NH + TFP; SH + TFP), with 0.25, 0.375, and 0.5 mM tBHP, respectively (NH + tBHP; SH + tBHP), or with 5 *μ*M TFP and 0.25, 0.375, and 0.5 mM tBHP, respectively (NH + TFP + tBHP; SH + TFP + tBHP). Cells were exposed to tBHP for a period of 30 min. The values are means ± SD (*n* = 16). ***P < 0.001 versus control NH; ^&&&^ *P* < 0.001 versus corresponding NH + tBHP group; ^###^ *P* < 0.001 versus control SH; ^xxx^ *P* < 0.001 versus corresponding NH + tBHP group; ^$$$^ *P* < 0.001 versus corresponding SH + tBHP group.

Steatotic hepatocytes, compared to nonfatty cells, exert significantly lower activity of cellular dehydrogenases (WST-1 test, Figure 1(a)) and almost 2-fold higher activity of LDH in culture medium (Figure 1(b)). Control fatty hepatocytes are also significantly more affected by oxidative stress as documented by higher production of ROS (Figures 1(c) and 3(a)) and MDA in culture medium (Figure 3(b)) and lower intracellular ratio of GSH to total glutathione (Figure 3(c)). There are no differences between control lean and steatotic hepatocytes in albumin production (Figure 2(c)), percentage of hepatocytes with energized mitochondria (Figures 4(a), 4(b), and 5), and oxygen consumption at state 4 respiration whereas oxygen consumption at state 3 respiration was significantly reduced in steatotic cells (Table 2).

### 3.2. Effect of tBHP on Nonfatty and Steatotic Hepatocytes

WST-1 test showed (Figure 1(a)) that lean hepatocytes exposed to tBHP for a period of 60 min are significantly affected from the concentration of 0.25 mmol/L, whereas in fatty hepatocytes, WST-1 test was already decreased at tBHP concentration of 0.1 mmol/L. Activity of LDH in culture medium did not exert any increase of LDH activity in nonsteatotic cells treated with 0.25 mM tBHP up to 60 min (Figure 1(b)). In fatty hepatocytes, LDH activity was significantly elevated even in 15 min after exposure to 0.25 mM tBHP. tBHP (0.25 and 0.375 mmol/L) induced more pronounced depression of albumin production in fatty hepatocytes, as compared to lean cells (Figure 2(c)).

Incubation with tBHP for 30 min revealed higher susceptibility of steatotic hepatocytes to oxidative stress. Generation of ROS after exposure to tBHP exerts dose and time dependent manner and is more pronounced in fatty hepatocytes (Figures 1(c) and 3(a)). Concentration of MDA in culture medium of fatty cells incubated with 0.25 mM tBHP was almost 2-fold higher than in lean hepatocytes (Figure 3(b)). tBHP at concentration of 0.25 mmol/L did not cause significant change in GSH to total glutathione ratio in nonsteatotic cells and incubation with 0.375 mM tBHP leads to only mild decrease by 5% in the ratio. In contrast, reduction in this ratio to 66% and 33% was observed in fatty hepatocytes exposed to tBHP at concentrations of 0.25 and 0.375 mmol/L, respectively (Figure 3(c)).

Figures 4(c), 4(d), 4(g), 4(h), and 5 show that steatotic hepatocytes exert higher susceptibility to tBHP-induced decrease in MMP. In lean cells, tBHP from concentration of 0.375 mmol/L leads to reduction of percentage of hepatocytes with energized mitochondria, whereas in fatty cells the reduction was more expressed and was found from tBHP concentration of 0.25 mmol/L.

Exposure to 0.25 mM tBHP for 5 min resulted in a nonsignificant increase in state 4 respiration by 24 and 26% in lean and fatty cells, respectively (compared with control nonsteatotic and steatotic hepatocytes, resp.). RCR of complex I and oxygen consumption at state 3 were reduced by tBHP in both lean and steatotic cells; NADH-dependent respiration at state 3 was significantly lower in fatty hepatocytes than in lean controls (Table 2).

### 3.3. Effect of TFP on Control Lean and Fatty Hepatocytes

Incubation of both lean and steatotic hepatocytes with 5 *μ*M TFP for a period of 60 min (30′ + 30′) did not lead to significant changes in LDH activity in culture medium (Figure 2(a)). TFP did not reduce activity of cellular dehydrogenases in nonsteatotic and fatty hepatocytes when compared with corresponding controls (Figure 2(b)). Exposure to TFP leads to the elevation of intracellular GSH to total glutathione ratio in control nonsteatotic hepatocytes and a similar, nonsignificant trend was also found in fatty cells (Figure 3(c)). In contrast to these beneficial effects, TFP alone significantly reduced production of albumin in both lean and steatotic cells (Figure 2(c)).

### 3.4. Potential Beneficial Effect of TFP on tBHP-Induced Injury

We compared two protocols of cell incubation with 5 *μ*M TFP. Firstly, hepatocytes were coincubated with tBHP and TFP for 30 min. Secondly, cells were at the earliest preincubated with TFP for a period of 30 min and subsequently coincubated with tBHP and TFP for additional 30 min. We found that TFP was able to partially reduce tBHP-induced damage (LDH activity and ROS production, data not shown) only when cells were firstly preincubated with TFP prior to tBHP exposure. Therefore we only present results with preincubation followed by coincubation.

TFP was able to partially prevent tBHP-induced elevation in LDH activity in culture medium of steatotic hepatocytes (Figure 2(a)), increase in production of ROS in both lean and fatty cells (Figure 3(a)), decrease in GSH/total glutathione ratio in nonfatty hepatocytes exposed to 0.375 tBHP (Figure 3(c)), and reduction in the percentage of cells with energized mitochondria in nonsteatotic hepatocytes exposed to 0.375 and 0.5 mM tBHP and in steatotic cells incubated with 0.25 and 0.375 mM tBHP (Figures 4 and 5). We did not observe any beneficial effect of TFP on activity of cellular dehydrogenases (Figure 2(b)), production of albumin (Figure 2(c)), and production of MDA (Figure 3(b)) in tBHP-treated nonfatty and steatotic hepatocytes.

## 4. Discussion

In this experiment, we studied oxidative stress-induced changes in hepatocytes isolated from nonfatty and steatotic rat liver. For evaluation of peroxidative damage, hepatocytes were exposed to tBHP, a prooxidant compound frequently used for assessment of mechanisms involving in oxidative stress in biological systems. Oxidative stress plays commonly a key role in the pathogenesis of both xenobiotic/drug-induced hepatotoxicity [27] and NAFLD [28]. We analysed the time course and the dose dependence of the peroxidative injury to hepatocytes induced by tBHP, and we correlated changes of cell viability, markers of oxidative stress (production of ROS, lipoperoxidation, and intracellular GSH content), the mitochondrial membrane potential, functional capacity of hepatocytes, and respiration of digitonin-permeabilized hepatocytes.

tBHP is known to cause peroxidation of membrane lipids [19] and deplete cellular GSH [20]. We and others have previously reported lower amounts of GSH in steatotic liver* in vivo* in patients [29] and in experimental models [21] and* in vitro *in mouse hepatocyte line (AML12 cells) treated with free fatty acids [30] or in rat hepatocytes isolated from fatty liver [17]. In contrast, induction of steatosis in the human liver cell line (HepG2/C3A) leads to elevation of cellular GSH [31]. Similarly, Grattagliano et al. [32] observed an early increase of liver GSH followed by its progressive decrease in a rat model of steatosis. This transient increment of GSH seems to be only a cellular adaptive antioxidant response to increased oxidative stress induced by excess of fat. Glutathione depletion is considered a potential biomarker of drug-induced hepatotoxicity. Moreover, tBHP is partially metabolized via glutathione peroxidase [20]; thus our observation of altered balance of intracellular glutathione redox state in steatotic cells predisposes to its toxicity and to susceptibility to oxidative stress in general. Decreased GSH to total glutathione ratio in control fatty hepatocytes is in a good concordance with findings of about twofold higher production of ROS and increased MDA production in these cells. tBHP-induced generation of ROS in lean and steatotic hepatocytes correlates well with the time of incubation and the dose of tBHP and is significantly more pronounced in fatty cells. Our study clearly showed that steatotic hepatocytes are more susceptible to oxidative injury caused by tBHP in primary culture than lean hepatocytes as documented by the activity of cellular dehydrogenases and LDH. In lean hepatocytes, we did not observe any damage to plasma membrane after incubation with 0.25 mM tBHP for up to 60 min. In contrast, LDH activity in the culture medium of fatty hepatocytes was significantly elevated even after cultivation with 0.25 mM tBHP for 15 min. Despite the same viability of lean and steatotic hepatocytes at beginning of the experiment (data not shown), plasma membrane integrity of control fatty cells was more disrupted than that of control lean cells. Altered redox balance and S-thiolation of crucial cellular components may be responsible for the inhibition of protein synthesis during the oxidative stress [33]. We showed that albumin production was reduced to 63% and 23% of control values in lean and steatotic hepatocytes, respectively, after 30 min incubation with 0.25 mM tBHP. Thus proteosynthetic function of hepatocytes seems to be more sensitive to oxidative stress in fatty hepatocytes.

Mitochondrial functions are often altered in the liver affected by NAFLD [5]. Thus induction of ROS production in the terrain of NAFLD leads to further progression of mitochondrial dysfunction with all consequences. Electron flow disruption at any point of the respiratory chain augments generation of ROS via transfer of electrons to molecular oxygen [8]. Besides other known mechanisms, ROS exert their toxicity also through the induction of MPT [34]. tBHP is known to induce MPT in isolated hepatocytes [35]. Although the onset of MPT was not directly measured in this study, we believe that JC-1-visualized changes of mitochondrial membrane potential (MMP) result from MPT. To support this statement, we treated hepatocytes with a MPT inhibitor. Since cyclosporin A is weakly effective in prevention of tBHP-induced cytotoxicity in hepatocytes, we used trifluoperazine which is known to specifically reduce MPT-mediated injury [35]. Broekemeier and Pfeiffer [36] suggested that TFP is able to increase the gating potential of the MPT pore and therefore block the MPT. TFP was capable of attenuating tBHP-induced decrease in MMP in both lean and steatotic hepatocytes. Thus changes of MMP are at least partially caused by MPT. Moreover, TFP reduced partially plasma membrane damage in steatotic hepatocytes, ROS production in both nonsteatotic and fatty cells, and altered GSH status in nonfatty hepatocytes exposed to tBHP. Similarly Shen et al. [37] also showed that MPT inhibitors may reduce superoxide mediated cytochrome c release and mitochondrial depolarization and subsequently inhibit apoptosis in HepG_2_ cells. Nevertheless, TFP was not significantly effective in prevention of reduced activity of cellular dehydrogenases, decreased albumin synthesis, enhanced lipoperoxidation, and surprisingly altered mitochondrial complex I activity (data not shown).

In steatotic liver, there is a higher offer of fatty acids to be peroxidized which together with an insufficient antioxidant capacity of the liver [29] leads to augmented lipoperoxidation. Here we proved that production of MDA in control fatty cells is twice as much that in lean cells. Additional exposure to external inducer of oxidative stress caused higher increase in MDA production in steatotic cells. Peroxidized lipids [38] together with ROS (i.e., superoxide anion) [39] belong to the activators of phospholipases A2. Since the involvement of free fatty acids, products of phospholipase A2 activity, in the triggering of MPT was shown [40], lipoperoxidation products and superoxide anion are thought to play an important role in this event in the environment of enhanced oxidative stress. TFP is also known to inhibit phospholipase A2 activity which may explain more effective prevention of tBHP-induced cytotoxicity in hepatocytes, in comparison with cyclosporine A. Bohm et al. showed that feeding rats corn oil containing peroxidized fatty acids may trigger the development of hepatic inflammation [41]. Thus, lipoperoxidation participates considerably in the pathogenesis of NAFLD and mediates progression from simple steatosis to advanced forms of NAFLD [42] with further increasing of oxidative stress.

Mitochondria, as the main energy provision system, is a crucial site of action of many hepatotoxic substances [12]. In the liver with accumulated fat, decreased activities of mitochondrial complexes I, II, IV, and V [21, 43] and elevated formation of ROS [3, 7, 10, 44] were detected. Mitochondrial dysfunction is characterized by permeabilization of mitochondrial outer membrane and resulting release of proteins from intermembrane space into the cytosol, caspase activation, disruption of the mitochondrial respiratory chain, loss of MMP, and augmented free-radical production [45]. Mitochondrial dysfunction is thought to represent a central abnormality responsible for progression from simple fatty liver to steatohepatitis [46]. Herein, we show respiration of digitonin-permeabilized rat hepatocytes. When observing complex I respiration, we found significant reduction in state 3 oxygen consumption and a trend of lower RCR in control fatty cells. In contrast to changes of complex I, Cardoso et al. reported trends of increased state 3 respiration and RCR of complex II (succinate as a substrate) in mitochondria isolated from liver of high-fat diet fed mice [44]. This is in agreement with our previous results showing that ADP-stimulated respiration using succinate together with NADH-linked substrates was not affected in steatotic permeabilized hepatocytes; thus flavoprotein-dependent substrates might compensate decreased activity of complex I [47]. Oxidative stress induced by tBHP reduces mitochondrial function in isolated hepatocytes [48]. We have previously proved that mitochondrial complex I is more sensitive to peroxidative damage of tBHP than complex II in nonfatty hepatocytes [26, 48]. Palmitoyl carnitine oxidation is strongly depressed by very low concentration of tBHP [49]; thus even mild oxidative stress leads to reduction of fatty acid oxidation by mitochondria and worsening of hepatocyte steatosis. Supply of succinate (a substrate of complex II) and inhibition of MPT by cyclosporine A may restore tBHP-induced decrease in MMP [26]. Our previous observations [47] and presented results show that respiratory complex I in permeabilized steatotic hepatocytes is even more susceptible to the effect of tBHP than in lean cells. Decreased activity of complex I substantially contributes to mitochondrial dysfunction by reducing the electron transport and the proton-motive force. In addition to reduced generation of ATP, dysfunction of respiratory complex I results in augmented production of superoxide anion [50]. Besides activation of apoptosis, loss of cytochrome c from intermembranous space leads to a dramatic increase in ROS generation and inhibition of respiration in mitochondria oxidising complex I substrates [50]. In our study, we observed a nonsignificant elevation of state 4 activity of complex I after exposure to tBHP in both lean and steatotic hepatocytes. Higher state 4 respiration may indicate tBHP-induced damage to the inner mitochondrial membrane and is proportionate to the rate of proton leakage across the inner membrane [51]. RCR was also considerably affected by the action of tBHP in both lean and fatty cells. The significant decrease of RCR induced by tBHP is caused by both the inhibition of ADP-dependent respiration and the elevation of state 4 respiration. Even mild inhibition (by 20%) of complex I activity, in contrast to inactivation of complex III, results in considerable increase in ROS production in mitochondria [52]. In addition to damage of complex I, lipoperoxidation further aggravates mitochondrial function. Byproducts of lipid peroxidation, such as MDA and 4-hydroxynonenal, are able to form adducts with cytochrome c oxidase and reduce its activity [53]. Oxidative stress induces mitochondrial dysfunction which causes an increase in ROS production and further injury to mitochondria.

## 5. Conclusion 

In summary, we demonstrated that there are higher production of ROS, increased lipid peroxidation, lower redox state of glutathione, and decreased ADP-stimulated respiration using NADH-linked substrates in control fatty hepatocytes, as compared to control lean hepatocytes. We provided evidence that steatotic rat hepatocytes isolated from fatty liver are more susceptible to oxidative injury caused by tBHP in primary culture. According to the partial effect of TFP in the prevention of tBHP induced injury, MPT seems to participate in the toxicity of tBHP in lean and steatotic hepatocytes and the onset of MPT appears to be caused by lower concentration of tBHP in fatty cells. In addition, the present study confirmed the significance of inhibition of complex I activity induced by tBHP in both lean and steatotic cells. Our results collectively indicate that steatotic rat hepatocytes in primary culture are under conditions of enhanced oxidative stress. Moreover, these fatty hepatocytes are more sensitive to the exogenous source of oxidative injury. Free radicals are not only a cause but also a consequence of human pathologies, such as NAFLD. Mitochondria play an essential role in the generation of ROS and at the same time mitochondria are an important target for toxic action of free radicals [54]. Our results confirm widely accepted hypothesis that steatosis is the first hit that sensitizes hepatocytes to further damage.

## Figures and Tables

**Table 1:** Scheme of protocol with 5 *μ*M TFP pretreatment.

Group of lean and steatotic hepatocytes\Time of incubation	0′–30′	30′–60′
Control	0	0
TFP	5 *μ*M TFP	5 *μ*M TFP
tBHP	0	tBHP
tBHP + TFP	5 *μ*M TFP	5 *μ*M TFP + tBHP

0: William's E medium without TFP or tBHP; TFP: trifluoperazine; tBHP: *tert *-butyl hydroperoxide at tested concentration.

**Table 2:** Respiration of digitonin-permeabilized (10 *μ*g/mL) hepatocytes (125,000/mL) in K^+^-medium at a temperature of 30°C. State 4 respiration (10 mM glutamate + 2.5 mM malate) and state 3 respiration (10 mM glutamate + 2.5 mM malate + 1.5 mM ADP) were measured and respiratory control ratio (RCR, state 3/state 4) was calculated in control nonsteatotic (NH) and steatotic rat hepatocytes (SH) and in lean and fatty hepatocytes preincubated with 0.25 mM tBHP for 5 min (NH + tBHP; SH + tBHP). Oxygen uptake at state 3 and state 4 is expressed as pmoles oxygen per second per million cells. **P < 0.01 and ***P < 0.001 versus NH; ^###^ *P* < 0.001 versus SH; ^$$^ *P* < 0.01 versus NH + tBHP.

	State 3	State 4	RCR of complex I
	(pmol O_2_/s/10^6^ cells)	(pmol O_2_/s/10^6^ cells)	
NH (*n* = 11)	1015.5 ± 145.1	188.5 ± 44.5	5.4 ± 1.2

NH + tBHP (*n* = 9)	761.0 ± 145.6	233.0 ± 61.6	3.3 ± 0.7
	∗∗∗	n.s. versus NH	∗∗∗

SH (*n* = 10)	819.7 ± 112.5	172.4 ± 42.5	4.8 ± 0.8
	∗∗	n.s. versus NH	n.s. versus NH

SH + tBHP (*n* = 8)	520.4 ± 98.2	217.6 ± 76.0	2.4 ± 0.6
	###	n.s. versus SH	###
	$$	n.s. versus NH + tBHP	n.s. versus NH + tBHP
